# Functional exercise capacity and health-related quality of life in people with asbestos related pleural disease: an observational study

**DOI:** 10.1186/1471-2466-13-1

**Published:** 2013-01-10

**Authors:** Marita T Dale, Zoe J McKeough, Phillip A Munoz, Peter Corte, Peter TP Bye, Jennifer A Alison

**Affiliations:** 1 Discipline of Physiotherapy (Rm0166) Faculty of Health Sciences, The University of Sydney, 75 East St Lidcombe, 2141, Sydney, NSW, Australia; 2 Physiotherapy Department, St Vincent’s Hospital, Sydney, NSW, Australia; 3 Department of Respiratory Medicine, Royal Prince Alfred Hospital, Sydney, NSW, Australia; 4 Sydney Medical School, The University of Sydney, Sydney, NSW, Australia; 5 Physiotherapy Department, Royal Prince Alfred Hospital, Sydney, NSW, Australia

**Keywords:** 6-minute walk test, Asbestos related diffuse pleural thickening, Exercise capacity, Physical activity, Quality of life

## Abstract

**Background:**

Functional exercise capacity in people with asbestos related pleural disease (ARPD) is unknown and there are no data on health-related quality of life (HRQoL). The primary aims were to determine whether functional exercise capacity and HRQoL were reduced in people with ARPD. The secondary aim was to determine whether functional exercise capacity was related to peak exercise capacity, HRQoL, physical activity or respiratory function.

**Methods:**

In participants with ARPD, exercise capacity was measured by the six-minute walk test (6MWT) and incremental cycle test (ICT); HRQoL by the St George’s Respiratory Questionnaire and physical activity by an activity monitor worn for one week. Participants also underwent lung function testing.

**Results:**

25 males completed the study with a mean (SD) age of 71 (6) years, FVC 82 (19)% predicted, FEV_1_/FVC 66 (11)%, TLC 80 (19)% predicted and D_L_CO 59 (13)% predicted. Participants had reduced exercise capacity demonstrated by six-minute walk distance (6MWD) of 76 (11)% predicted and peak work rate of 71 (21)% predicted. HRQoL was also reduced. The 6MWD correlated with peak work rate (r=0.58, p=0.002), St George’s Respiratory Questionnaire Total score (r=-0.57, p=0.003), metabolic equivalents from the activity monitor (r=0.45, p<0.05), and FVC % predicted (r=0.52, p<0.01).

**Conclusions:**

People with ARPD have reduced exercise capacity and HRQoL. The 6MWT may be a useful surrogate measure of peak exercise capacity and physical activity levels in the absence of cardiopulmonary exercise testing and activity monitors.

**Trial registration:**

ANZCTR12608000147381

## Background

Asbestos related pleural disease (ARPD) is a worldwide problem with non-malignant pleural disease a common manifestation of asbestos exposure. Despite tighter regulations in the use of asbestos in many developed countries, the legacy of asbestos exposure remains and the incidence of asbestos-related pleural abnormalities continues to rise.

Asbestos related pleural disease may result in pleural fibrosis [1]. Despite being recognized as a separate entity to pulmonary fibrosis [2], ARPD remains poorly investigated and understood. The ensuing symptoms, such as shortness of breath on exertion [3,4] are similar to other chronic respiratory diseases and may cause considerable functional impairment to the individual. Previous studies have demonstrated abnormal responses or reductions in peak exercise capacity during cardiopulmonary exercise testing [5-7] in people with ARPD. However, no studies have investigated the effects of ARPD on functional exercise capacity.

The six-minute walk test (6MWT) is a measure of functional exercise capacity widely used in the assessment of lung diseases including chronic obstructive pulmonary disease (COPD) [8]. Evidence is growing for the value of the 6MWT in evaluating functional exercise capacity in interstitial lung diseases [9] yet functional exercise capacity in people with ARPD is unknown.

The effects of ARPD on health-related quality of life (HRQoL) have not previously been investigated. Chronic respiratory diseases are frequently associated with decrements in HRQoL [10]. Furthermore, there are no data on the effects of ARPD on levels of physical activity. Levels of physical activity in other lung diseases, such as COPD, have been linked to health outcomes and HRQoL [11,12].

The primary aims of this study were to determine whether functional exercise capacity and HRQoL were reduced in people with ARPD. The secondary aim was to determine whether functional exercise capacity was related to peak exercise capacity, HRQoL, physical activity or respiratory function.

## Methods

### Subjects

This observational study was conducted at Royal Prince Alfred Hospital, Sydney, Australia from November 2008 - August 2010. Participants were recruited through the Workers’ Compensation Dust Diseases Board (DDB) of New South Wales, respiratory physicians, support groups, workers’ unions and newsletters for returned servicemen.

People were eligible to participate if they had a diagnosis of ARPD, defined as asbestos-related diffuse pleural thickening and/ or rounded atelectasis. Diagnosis had been established by the participant’s respiratory physician or the DDB Medical Authority, a panel of three respiratory physicians with specialist knowledge in occupational lung disease. The diagnostic process at the DDB has previously been described, and includes radiological investigation, lung function testing, clinical examination by a thoracic physician and a lifetime occupational history [13]. Computerised tomography (CT) scans had been conducted on all participants prior to study commencement.

People were excluded from the study if they had mesothelioma; discrete parietal pleural plaques as their only manifestation of dust exposure; cardiovascular, neurological or orthopaedic conditions limiting exercise performance; were on long term oxygen therapy; could not understand English; or had participated in pulmonary rehabilitation within the last 12 months.

The study was approved by the Human Research Ethics Committee of Sydney South West Area Health Service. All participants gave written informed consent.

### Pulmonary function tests

Participants performed pulmonary function tests of spirometry, lung volumes (body plethysmography) and single breath diffusing capacity for carbon monoxide (D_L_CO) (SensorMedics, Yorba Linda, Ca, USA). Tests were performed according to American Thoracic Society (ATS) guidelines and results expressed as a percentage of predicted values [14-16]. Maximal voluntary ventilation (MVV) was calculated as forced expiratory volume in one second (FEV_1_) multiplied by 40 [17]. Forced vital capacity (FVC) and D_L_CO were the pulmonary function values used in the correlation analyses.

### Exercise testing

Participants performed two 6MWTs (6MWT 1 and 6MWT 2) according to ATS guidelines [18] on a 32-metre oval track with tests separated by a minimum of 30 minutes. Throughout both tests standardised instructions and encouragement were given. Each minute pulse rate (PR) and oxygen saturation (SpO_2_) were measured (Radical™, Masimo Corporation, Irvine, USA) and dyspnoea and rate of perceived exertion (RPE) scores ranging from 0-10 (0 was ‘nothing at all’ and 10 was ‘maximal’) were recorded [19,20]. The better 6MWT was used for analysis.

On a second day of testing, participants performed a symptom-limited incremental cycle test (ICT) to peak work capacity on an electromagnetically-braked cycle ergometer (Lode BV, Groningen, The Netherlands). Following two minutes of rest and one minute of unloaded cycling, work rate was increased every minute by a predetermined amount, from 5 to 20 W.min^-1^ according to the participant’s self-reported exercise capacity and disease severity so that the test was approximately 10 minutes duration [21]. Breath-by-breath values for oxygen uptake (VO_2_) and carbon dioxide output (VCO_2_) were obtained (Vmax Encore, SensorMedics, Yorba Linda, USA). Volume and gas calibration were performed prior to each test. Pulse rate and SpO_2_ were simultaneously measured and dyspnoea and RPE scores were recorded each minute and at peak work rate. The test was ceased when the participant reached symptom-limited maximum. Results of the 6MWT and ICT were compared to predicted normal values [22,23].

### Health-related quality of life (HRQoL)

Participants completed the St George’s Respiratory Questionnaire (SGRQ) [24]. A priori, the ‘Activity’ domain and ‘Total’ score from the SGRQ were identified to examine against measures of exercise capacity.

### Physical activity

Participants wore an activity monitor (SenseWear Pro3 Armband, BodyMedia, Pittsburgh, PA, USA) for one-week when not attending exercise testing. Participants were instructed to wear the armband continuously, removing it only when showering or swimming. The activity monitor, worn on the right triceps, incorporated a biaxial accelerometer and sensors for skin temperature, heat flux, and galvanic skin resistance. Mean data on steps per day, the daily metabolic equivalents (METs) and energy expenditure were recorded. A minimum compliance of three days of wear with a daily compliance level of 85% was specified for increased measurement accuracy [25]. If this level of compliance was not achieved, the data or day was excluded from analysis.

### Statistical analysis

Statistical analysis was performed on PASW-Windows (release 18.0; PASW, Chicago, IL, USA). Data are expressed as mean (SD) or (95% CI). A paired-sample *t* test was used to compare the distance walked in 6MWT 1 and 6MWT 2, and to compare dyspnoea, PR and RPE achieved in the better 6MWT with those from the ICT. Relationships between variables were examined using Pearson’s correlation coefficients. The level of significance was set at a *p*-value of <0.05.

## Results

### Subjects

Twenty-eight male participants were assessed with 25 included in the study. The reasons for non-inclusion of three participants were pain affecting exercise performance (one), neurological impairment (one) and a significant degree of emphysema (one). Mean anthropometric data and pulmonary function are shown in Table 1.

**Table 1:** Demographic data, pulmonary function and smoking history

	**n**=**25 mean** (**SD**)
Age, yr	71 (6)
Height, cm	174 (5)
Weight, kg	84 (12)
BMI, kg/m^2^	28 (3)
FVC, % pred	82 (19)
FEV_1_, % pred	74 (20)
FEV_1_/FVC %	66 (11)
TLC, % pred	80 (19)
FRC, % pred	80 (23)
RV, % pred	78 (29)
D_L_CO, % pred	59 (13)
KCO, % pred	84 (18)
Smoking, pack year	12 (15)
Never smoked (n)	6

*n* = number; *SD* = standard deviation; *yr* = year; *cm* = centimetre; *kg* = kilogram; *BMI* = body mass index; m = metre; *FVC* = forced vital capacity; % pred = percentage of predicted value; *FEV*_*1*_ = forced expiratory volume in one second; *TLC* = total lung capacity; *FRC* = functional residual capacity; *RV* = residual volume; *D*_*L*_*CO* = diffusing capacity for carbon monoxide; *KCO* = carbon monoxide transfer coefficient.

### Exercise capacity

Participants demonstrated reduced functional exercise capacity measured by the 6MWT when compared to predicted values [22] (Table 2). Participants also demonstrated reduced peak work capacity. The subjective reasons for ceasing the ICT were dyspnoea in eight participants, leg fatigue in ten participants and combined dyspnoea and leg fatigue in seven participants.

**Table 2:** Exercise test results for the 6MWT and the ICT, health-related quality of life and physical activity data

	**mean (SD)**
**6MWT**	**n**=**25**
6MWD, m	486 (68)
6MWD, % pred	76 (11)
Resting SpO_2_, %	97 (1)
Desaturation, %	4 (3)
Peak PR, % pred*	67 (11)
Dyspnoea at test end	2 (2)
RPE at test end	1 (2)
**ICT**	**n**=**25**
Work rate_peak_, W	114 (36)
Work rate_peak_, % pred	71 (21)
VO_2_ peak, % pred	83 (22)
VE, L/min	54 (14)
VE/MVV	63 (16)
Resting SpO_2_, %	99 (1)
Desaturation, %	1 (2)
PR, % pred	82 (13)
Dyspnoea at peak	4 (2)
RPE at peak	5 (2)
**HRQoL**	**n**=**25**
SGRQ Symptoms	29 (21)
SGRQ Activity	34 (22)
SGRQ Impacts	15 (14)
SGRQ Total	23 (15)
**Physical activity**	**n**=**23**
Average daily steps	9072 (3186)
Average daily METs	1.3 (0.2)
Average daily EE, cal	2630 (459)

*SD* = standard deviation; *6MWT* = six-minute walk test; *n* = number; *6MWD* = six-minute walk distance; *m* = metre; *% pred* = percentage of predicted value; *SpO*_*2*_ = oxygen saturation; *PR* = pulse rate; *RPE* = rate of perceived exertion; *ICT* = incremental cycle test; *W* = watts; VO_2_peak % pred = percent of predicted normal maximal oxygen uptake; *VE* = minute ventilation; *MVV* = maximal voluntary ventilation; *HRQoL* = health-related quality of life; *SGRQ* = St George’s Respiratory Questionnaire; *METs* = metabolic equivalent; *EE* = energy expenditure; *cal* = calorie.

* Predicted PR = 220 - age.

### Health-related quality of life

Participants demonstrated reduced levels of HRQoL across all domains of the SGRQ. Mean data are shown in Table 2.

### Repeatability of the six-minute walk test

There was a significant mean difference between 6MWT 1 and 6MWT 2 of 13 metres (95% CI: 6 to 21) (*p*<0.001) with 80% of participants walking further on 6MWT 2.

### Responses during the better 6MWT

**Figure 1:**
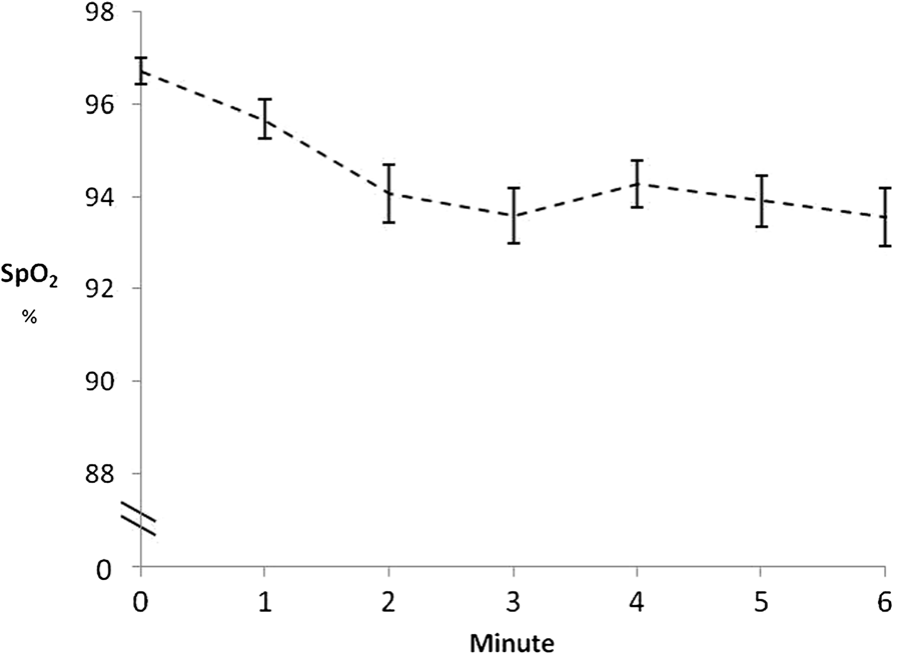
**SpO**_**2 **_**response during the 6MWT.** SpO_2_ = oxygen saturation; Error bars = Standard error.

**Figure 2:**
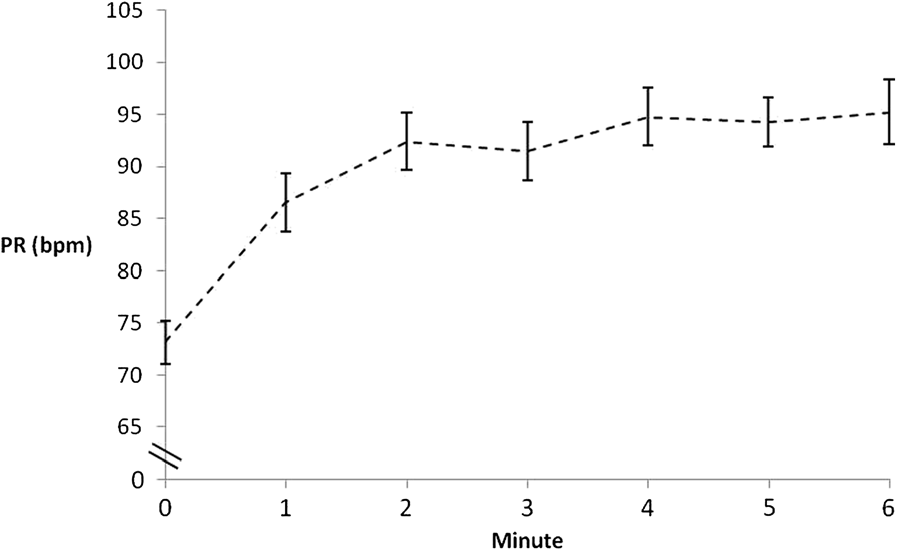
**Pulse rate response during the 6MWT.** PR = pulse rate; bpm = beats per minute; Error bars = Standard error.

In the better 6MWT, 44% of participants desaturated by ≥ 4%. There was a significantly greater desaturation during the 6MWT compared to the ICT, mean difference 3% (95% CI: 1 to 4), (*p*<0.001). The PR, RPE and dyspnoea responses at the end of the 6MWT were significantly lower than at the end of the ICT (Table 3). The minute-by-minute SpO_2_ and PR responses during the 6MWT are shown in Figures 1 and 2.

**Table 3:** Mean difference in end exercise PR, dyspnoea and RPE between ICT and 6MWT

**Variable**	**ICT mean** (**SD**)	**6MWT mean** (**SD**)	**Mean difference** (**95%CI**)	***p***
PR, b/min	122 (19)	100 (15)	21 (14 to 28)	<0.001
Dyspnoea, score	4 (2)	2 (2)	3 (2 to 4)	<0.001
RPE, score	5 (2)	1 (2)	4 (3 to 5)	<0.001

*ICT* = incremental cycle test; *6MWT* = six-minute walk test; *SD* = standard deviation; *CI* = confidence interval; *PR* = pulse rate; *b/min* = beats per minute; *RPE* = rate of perceived exertion.

### Relationships between functional and peak exercise capacity

There were significant positive correlations between 6MWD and peak work rate, r=0.58 (*p*=0.002) and between 6MWD and VO_2_ peak (ml/kg/min), r=0.53 (*p=*0.006).

### Physical activity

The activity monitor data was unavailable for two participants who did not wear it for the minimum three days due to skin irritation. Among remaining participants, the activity monitor was worn for a mean (SD) of 6 (1) days with a mean compliance of 98 (1)%. Physical activity data are presented in Table 2.

### Relationships of exercise capacity to lung function, physical activity and health-related quality of life

The 6MWD was significantly correlated with pulmonary function, physical activity and HRQoL (Table 4).

**Table 4:** Relationships between 6MWD or peak work rate and HRQoL scores, pulmonary function tests and measures of physical activity (r-values)

**Variable**	**ARPD n**=**25**	
	**6MWD**	**Peak work rate**
FVC, % pred	0.52^†^	0.59^†^
D_L_CO, % pred	0.50^‡^	0.64^*^
Daily steps	0.38	0.24
Daily METs	0.45^‡^	0.22
Daily energy expenditure	0.06	0.25
SGRQ Total	−0.57^†^	−0.71^*^
SGRQ Activity	−0.50 ^‡^	−0.70^*^

*p <0.001, ^†^*p* <0.01, ^‡^*p*<0.05.

*ARPD* = asbestos related pleural disease; *n* = number; *6MWD* = six-minute walk distance; *FVC* = forced vital capacity; *% pred* = percentage of predicted value; *D*_*L*_*CO* = diffusing capacity for carbon monoxide; *METs* = metabolic equivalent; *SGRQ* = St George’s Respiratory Questionnaire.

## Discussion

This study examined the effects of ARPD on functional exercise capacity and HRQoL. The main findings were people with ARPD had reduced functional exercise capacity and HRQoL, despite only having pleural involvement. In addition, this study showed significant relationships of functional exercise capacity to peak exercise capacity, physical activity and HRQoL in people with ARPD. The relationships demonstrated that a lower 6MWD was significantly associated with a lower peak work rate, level of physical activity and HRQoL. Such findings have not been previously demonstrated.

Functional exercise capacity and peak exercise capacity were reduced compared to predicted values. At peak exercise, the limiting symptom was leg fatigue in 40% of participants and dyspnoea in 32% of participants. Leg fatigue at exercise levels below predicted peak may indicate peripheral deconditioning [26] whereas dyspnoea as the limiting symptom may be attributable to decreased chest wall compliance caused by diffuse pleural thickening [5]. These findings differ from a study in people with idiopathic pulmonary fibrosis (IPF), which reported that 35% of participants stopped exercise due to leg fatigue and 65% due to dyspnoea [27]. This difference is likely due to greater disease severity in the IPF group compared to the people with ARPD, but also may be attributable to the slightly older age of our participants. The higher prevalence of leg fatigue may be associated with skeletal muscle changes attributable to aging [28], resulting in greater peripheral deconditioning and earlier onset of leg fatigue.

Health-related quality of life was reduced in people with ARPD as measured by the SGRQ domains. Despite these reductions, people with ARPD had higher levels of HRQoL than reported in people with IPF [29] and COPD [30]. To our knowledge, this is the first study to demonstrate that people with ARPD experience reductions in HRQoL.

In people with ARPD we have demonstrated a moderate relationship between 6MWD and peak work rate, although weaker than relationships in COPD (r=0.63, *p*<0.002; r=0.75, p<0.001)) [31,32]. We have also demonstrated a moderate relationship between 6MWD and VO_2_peak (ml/kg/min), although weaker than in end-stage lung diseases (r=0.73, *p*<0.001) [33]. This is likely due to the lesser disease severity in our participants. Despite this, the relationship between 6MWD and peak exercise capacity suggests that the 6MWT may be a useful surrogate measure of peak exercise capacity in people with ARPD when cardiopulmonary exercise testing is unavailable.

The 6MWT correlated moderately with the SGRQ Total score and the SGRQ Activity domain score, similar to relationships reported in COPD and interstitial lung disease [24,29]. This suggests the impact of ARPD on HRQoL may be reflected by the SGRQ, a questionnaire originally designed for people with COPD.

Physical activity differs from exercise capacity. Higher levels of daily physical activity have health benefits for people with COPD [34] or coronary artery disease [35]. Conversely, reduced physical activity is related to increased morbidity and mortality in COPD [11]. In this study, the 6MWD correlated more strongly with daily METs than did peak work rate, demonstrating the 6MWD may better reflect daily physical activity than a peak exercise test. This is likely the consequence of daily activities being performed at sub-maximal levels of intensity, rather than maximal levels of intensity [18]. In the absence of activity monitors in the clinical setting, the 6MWT may be a useful surrogate measure of physical activity.

There was a significant increase in distance walked between the first and second 6MWT of 13 metres or 3%, a smaller increase than reported in COPD [36] and interstitial lung disease [9]. In COPD, two 6MWTs are recommended to obtain an accurate measure of functional exercise capacity. The small increase in distance walked in the second 6MWT in people with ARPD questions whether repeat testing is clinically important in this population. However, functional exercise capacity may be underestimated if a second test is not performed.

Participants demonstrated a greater arterial oxyhaemoglobin desaturation during the 6MWT compared to the ICT, similar to people with COPD, and related to the larger exercising muscle mass utilised during the 6MWT [31]. We have demonstrated a peak cycle test is not required to examine arterial oxyhaemoglobin desaturation in people with ARPD and the 6MWT may provide valuable and unique information on oxygen desaturation during exercise*.*

The development of ARPD is often characterized by a long latency period from exposure to dust to development of disease [37]. As a result, the mean age of participants was 71 years. With increasing age, the FEV_1_/FVC ratio is known to decrease and the FEV_1_/FVC ratio in our participants was within the range of predicted normal values for people of this age [14].

This study has some limitations. No data were collected on the metabolic and ventilatory responses to the 6MWT so no direct comparisons can be made for these outcomes with the ICT. People on long term oxygen therapy were excluded so the findings of this study cannot be extrapolated to such patients. We did not exclude people if they had a smoking history as this would be unrepresentative of the patient population. Finally, we did not have a group of healthy aged-matched controls upon which a statistical comparison could be made, however data were compared to previously published predicted values for functional and peak exercise capacity.

## Conclusions

This is the first investigation of the effect of ARPD on functional exercise capacity, demonstrating that this population has reduced functional exercise capacity measured by the 6MWT. This study has also established that people with ARPD have reduced HRQoL. Furthermore, we have shown the 6MWD correlated with peak exercise capacity, HRQoL and physical activity. The 6MWT would be a simple test to perform and integrate into clinical practice to determine functional exercise capacity in people with ARPD and may be a useful surrogate measure of peak exercise capacity and physical activity in the absence of cardiopulmonary exercise testing and activity monitors. With few treatment options available for people with ARPD, research is required to address whether the impairments of reduced exercise capacity and HRQoL are amenable to pulmonary rehabilitation.

## Abbreviations

ARPD: Asbestos related pleural disease;6MWT: Six-minute walk test;COPD: Chronic obstructive pulmonary disease;HRQoL: Health-related quality of life;DDB: Dust Diseases Board;CT: Computerised tomography;DLCO: Diffusing capacity for carbon monoxide;ATS: American Thoracic Society;MVV: Maximal voluntary ventilation;FEV1: Forced expiratory volume in one second;FVC: Forced vital capacity;PR: Pulse rate;SpO2: Oxygen saturation;RPE: Rate of perceived exertion;ICT: Incremental cycle test;VO2: Oxygen uptake;VCO2: Carbon dioxide output;SGRQ: St George’s Respiratory Questionnaire;MET: Metabolic equivalent;SD: Standard deviation;CI: Confidence interval;IPF: Idiopathic pulmonary fibrosis

## Competing interests

The authors declare that they have no competing interests.

## Authors’ contributions

MD: study design, data collection, data analysis and interpretation, writing of the manuscript. ZM: study design, data collection, data analysis and interpretation, writing of the manuscript. PM: data collection and writing of the manuscript. PC: study design and writing of manuscript, PB: study design and writing of manuscript, JA: study design, data collection, data analysis and interpretation, writing of the manuscript. All authors read and approved the final manuscript.

## Support

Workers’ Compensation Dust Diseases Board (DDB) of New South Wales.

## Pre-publication history

The pre-publication history for this paper can be accessed here:

http://www.biomedcentral.com/1471-2466/13/1/prepub
